# Phytochemical investigation of *Magnolia grandiflora* green seed cones: Analytical and phytoceutical studies

**DOI:** 10.1002/fsn3.1016

**Published:** 2019-04-14

**Authors:** Bo Garza, Alondra Echeverria, Felipe Gonzalez, Orlando Castillo, Thomas Eubanks, Debasish Bandyopadhyay

**Affiliations:** ^1^ Department of Chemistry The University of Texas Rio Grande Valley Edinburg Texas; ^2^ School of Earth Environment and Marine Sciences (SEEMS) The University of Texas Rio Grande Valley Edinburg Texas

**Keywords:** druggability, gas chromatography‐mass spectroscopy, *Magnolia grandiflora*, phytoceutical, phytochemical, X‐ray crystallography

## Abstract

Phytochemicals are inevitable part of human civilization. It is impossible to say exactly when menfolk started to take plant portions to cure various diseases. Phytochemical investigation of diethyl ether and ethanol extracts of *Magnolia grandiflora* green seed cones has been carried out. Extraction, isolation, and identification of the phytochemicals were carried out. Structures were determined by various analytical methods including extensive nuclear magnetic resonance, gas chromatography‐mass spectroscopy, and X‐ray crystallographic analyses. Structures of the three compounds *viz*. 5,5′‐diallyl‐[1,1′‐biphenyl]‐2,2′‐diol (**I**), 3′,5‐diallyl‐[1,1′‐biphenyl]‐2,4′‐diol (**II**), and (3*S*,3*aS*,8*S*,9*aS*,10*aR*,10*bS*,*E*)‐8‐hydroxy‐3,6,9*a*‐trimethyl‐3*a*,4,5,8,9,9*a*,10*a*,10*b*‐octahydrooxireno[2′,3′:9,10]cyclodeca[1,2‐*b*]furan‐2(3*H*)‐one (**III**) were confirmed by X‐ray crystallographic analysis. GS‐MS studies of the isolated oil, eluted with hexanes, revealed the presence of eight compounds including two highly bio‐privileged molecules 5,5′‐diallyl‐2′‐methoxy‐[1,1′‐biphenyl]‐2‐ol (**IV**) and 1‐(4‐isopropylbenzyl)‐1,3‐dihydro‐2*H*‐benzo[*d*]imidazol‐2‐one (**V**). The druggability of the five compounds has also been determined by in silico studies. The isolated compounds and/or their semi‐synthetic products may find application in natural drug development research.

## 1 INTRODUCTION

**Figure 1:**
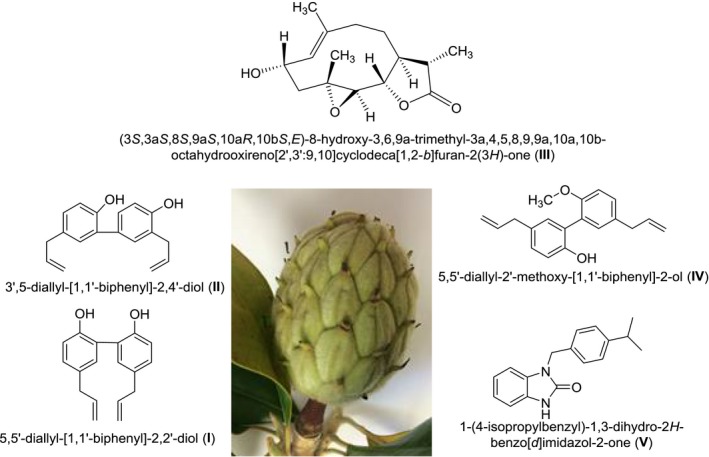
Compounds obtained from *Magnolia grandiflora *green seed cones

Medicinal plants are being used for thousands of years as remedies for human ailments because of the presence of numerous phytoceuticals. In practice, the term “phytochemicals” refers to a wide variety of compounds that are found in plants and validate health benefits on human/animal. “Phyto” is a Greek word that indicates “plant.” Accordingly, chemicals that are derived from plants are called “phytochemicals.” Phytochemicals can be broadly categorized into five major categories such as phytoceuticals, nutraceuticals, addictives (narcotics), toxins, and biologically inactive compounds. Out of these five categories, phytoceuticals and nutraceuticals are pharmacologically/medicinally relevant molecules that produce therapeutic effects (positive health effect); addictives have negative health effects, whereas toxins cause fatal health effect (Bandyopadhyay, 2014a,2014b). The utilization of natural products and/or their novel cores, in order to discover and develop the final drug entity, is still an interesting and highly promising area of drug discovery research. For example, in the area of cancer, from around the 1940s to the end of 2014, 175 small molecules were approved by the US Federal Drug Administration (FDA) or its similar organizations worldwide. Out of these 175 small molecule anticancer drugs, 131 (74.85%) are completely *nonsynthetic* molecules. Presently, about 25% of medicines are directly derived from nature. About 61% of new chemical entity (NCE) can be traced to a natural product origin. In certain therapeutic areas, this impact is even higher. For example, about 75% of anticancer drugs and 78% of antibacterial drugs are either natural products or chemically modified natural products or semi‐synthetic natural products. The rapidly evolving recognition that a significant number of drugs/leads/hits are produced by Mother Nature, and therefore it is considered that this area of natural resources should be expanded significantly (Giddings & Newman, 2015a,2015b,2015c; Newman & Cragg, 2016). Accordingly, chemical investigation of natural compounds is one of the most reliable and traditional routes to discover new and novel natural drugs. Magnolias (belong to the family *Magnoliaceae*) are known worldwide for their beautiful flowers with intense fragrance. Many species of the genus *Magnolia* are grown around the world, and out of all these different species, *Magnolia grandiflora* is the most attractive one because of its large size, gorgeous color, and fragrance (Avonto, Chittiboyina, Sadrieh, Vukmanovic, & Khan, 2018; Ding et al., 2018; Lata et al., 2017). This article deals with the phytochemical investigation of the diethyl ether and ethanol extracts derived from the *Magnolia grandiflora* green seed cones. Along with other compounds, five medicinally privileged compounds have been isolated and identified as drug‐like molecules through in silico evaluation. Appropriate chemical modifications of these molecules aiming to synthesize suitable semi‐synthetic compounds for drug–protein interactions might lead to develop novel therapeutics. These compounds (Figure 1) were characterized through various analytical techniques including gas chromatography‐mass spectroscopy (GC‐MS) and X‐ray crystallographic analyses.

## 2 MATERIALS AND METHODS

### 2.1 General experimental

Melting points were determined in a DigiMelt MPA 160 (Stanford Research Systems, USA) digital melting point apparatus. FT‐IR spectra were recorded on a Bruker Alpha modular Platinum‐ATR FT‐IR spectrometer with OPUS software, using the samples directly (neat) without making pallets. ^1^H‐NMR (600 MHz) and ^13^C‐NMR (150 MHz) spectra were obtained at room temperature with Bruker superconducting Ultrashield Plus 600 MHz NMR spectrometer with central field 14.09 T, coil inductance 89.1 H, and magnetic energy 1,127.2 kJ using CDCl_3_ or d_6_‐DMSO as solvent. The GC‐MS analysis of the oil was carried out by MassHunter gas chromatography‐mass spectrometer (Agilent Technologies, Inc.) using the MS firmware version 7.02.22. The column information is as follows: column‐HP‐5MS UI; length = 30 meters; diameter = 0.25 (mm); film thickness = 1.00 (μm); carrier gas was helium. X‐ray crystallographic studies were conducted in SuperNova, Dual, Cu at zero, Atlas diffractometer with Mo *K*α radiation (λ = 0.71073 Å) under the program CrysAlisPro (Version 1.171.36.32 Agilent Technologies, 2013). The same program was used to refine the cell dimensions and for data reduction. The computer programs used were *CrysAlis PRO*, Agilent Technologies, version 1.171.36.32 (release 02–08‐2013 CrysAlis171.NET) (compiled Aug 02 2013), *SHELXS2014*/7, *SHELXL2014*/7 (Sheldrick, 2015), and *SHELXTL* v6.10 (Sheldrick, 2008). All the solvents were purchased from Fisher‐Scientific throughout the investigation. Deionized water was used for the preparation of all aqueous solutions.

### 2.2 Plant materials

Fresh and mature green seed cones were collected directly from a *Magnolia grandiflora* tree located at the Edinburg campus of the University of Texas Rio Grande Valley and authenticated by Dr. Andrew McDonald, Professor of Ethnobotany, Department of Biology of the University of Texas Rio Grande Valley.

### 2.3 Extraction, fractionation, and separation

Bioactivity‐guided extraction was performed (Bandyopadhyay, Banerjee, Laskar, & Basak, 2011; Bandyopadhyay et al., 2006, 2007; Scoffoni et al., 2017). Fresh mature seed cones were chopped into small pieces and air‐dried in absence of sunlight and grinded to produce powder (679 g). The powder was submerged in diethyl ether at room temperature for 21 days. After the stipulated period, the solvent was removed from the extract under reduced pressure. The resultant gummy mass was chromatographed over silica gel and eluted with hexanes/ethyl acetate mixtures by increasing the polarity gradually. A reddish oil was isolated from the pure hexanes eluent. The hexanes/ethyl acetate (95:5) mixture afforded a reddish solid, which on recrystallization over dichloromethane/hexanes/diethyl ether gave reddish needle‐shaped crystals (1.67 g) of 5,5′‐Diallyl‐[1,1′‐biphenyl]‐2,2′‐diol (**1**). Another compound, 3′,5‐diallyl‐[1,1′‐biphenyl]‐2,4′‐diol (**II**), was collected from the same column using hexanes/ethyl acetate (93:7) mixture as eluent which on crystallization yielded (623 mg) white crystals of (**II**). The residue obtained from the diethyl ether extraction was re‐extracted with ethanol following the same procedure and the crude extract was chromatographed. The compound 2α‐hydroxydihydroparthenolide, a sesquiterpenoid lactone, was collected from the hexanes/ethyl acetate (40:60) mixture as eluent which on crystallization yielded (71 mg) light violet crystals of (**III**).

## 3 RESULTS AND DISCUSSION

The melting points of the compounds (**1**), (**II**), and (**III**) were recorded as 102–103°C, 87–88°C, and 220–221°C, respectively. Both 1D‐ and 2D‐NMR experiments were conducted to determine the structure of the compound. APT (Attached Proton Test), DEPT (Distortionless Enhancement of Polarization Transfer)‐45° and 135° experiments were carried out to reveal the nature of the carbons (methyl, methylene, methine, and quaternary) in the molecule. ^1^H–^1^H‐correlations were figured out by double quantum filtered correlation spectroscopy (DQF‐COSY). Short range and long range ^1^H–^13^C‐correlations were determined by HMQC (Heteronuclear Multiple‐Quantum Correlation) and HMBC (Heteronuclear Multiple Bond Correlation) experiments, respectively. The analytical data of the compounds (**I**), (**II**), and (**III**) are presented in the sequel.

### 3.1 Characterization of compound (**I**)

5,5′‐Diallyl‐[1,1′‐biphenyl]‐2,2′‐diol (**I**): Mp 102–103°C; IR (neat, ν in cm^−1^): 3,132, 2,994, 1,493, 1,408, 1,209, 1,155, 1,089, 989, 901, 817, 661; ^1^H‐NMR (600 MHz, CDCl_3_) δ 7.09 (d, *J = *8.04 Hz, 2H), 7.07 (s, 2H), 6.90 (dd, *J = *10.56, 2.22 Hz, 2H), 6.02 (s, 2H), 5.95 (m, 2H), 5.07 (m, 4H), 3.35 (d, *J = *6.72 Hz, 2H); ^13^C‐NMR (150 MHz, CDCl_3_) δ 151.98, 137.55, 133.32, 131.35, 129.91, 124.17, 116.75, 115.86, 39.39.

**Figure 2:**
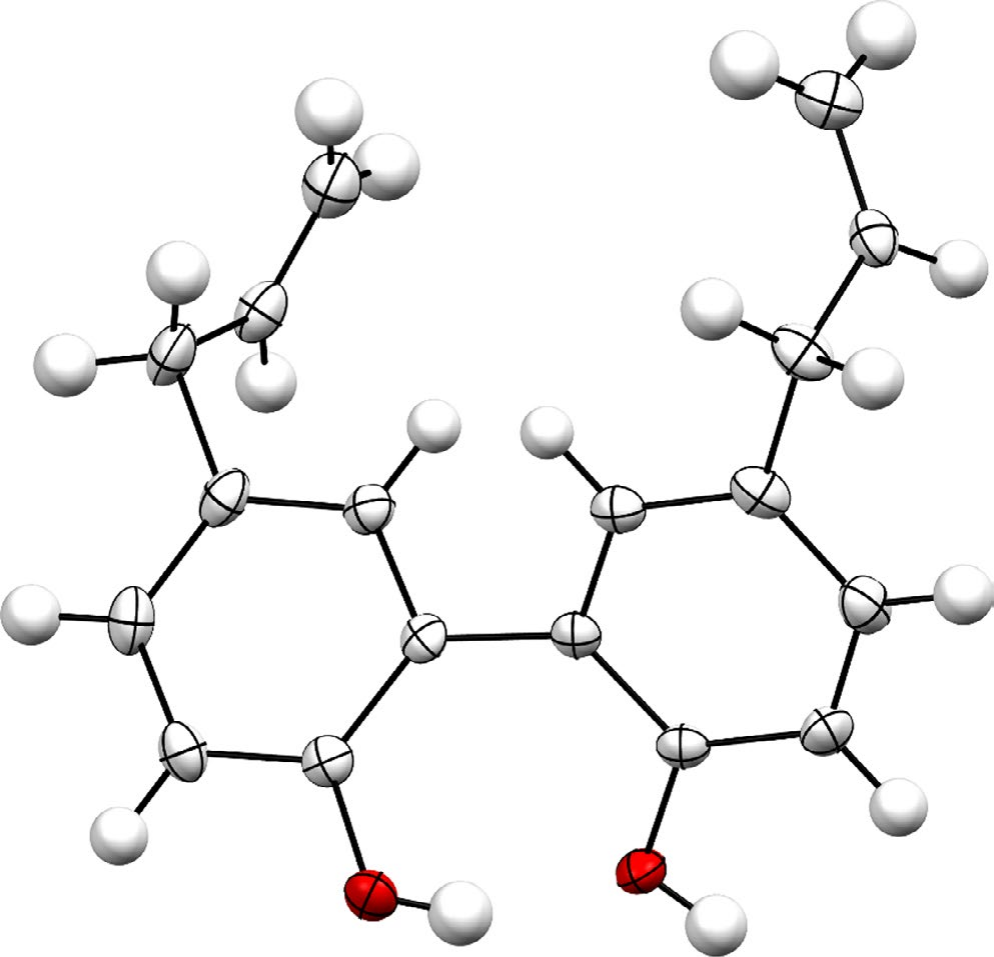
X‐ray crystallographic analysis of compound (**I**)

In FT‐IR spectrum, the broad peak at 3,132 cm^‐1^ identified the phenolic O–H stretching, whereas the presence of terminal double bonds was determined by 1D‐NMR experiments. The structure of the compound (**I**) was further confirmed by X‐ray crystallographic analysis. The ORTEP projection of the compound (**I**) is presented in Figure 2.

The structure was solved with the program SHELXS‐2014/7 and was refined on *F^2^* with SHELXL‐2014/7 (Sheldrick, 2015). Numerical absorption correction based on Gaussian integration over a multifaceted crystal model was applied using CrysAlisPro. The temperature of the data collection was controlled using the system Cryojet (manufactured by Oxford Instruments). The H atoms were placed at calculated positions using the instructions AFIX 23, AFIX 43, AFIX 93, or AFIX 137 with isotropic displacement parameters having values 1.2 or 1.5 *U*eq of the attached C atoms. The structure is ordered and the details are shown in Table 1.

**Table 1:** X‐ray crystallographic details of the compound (**I**)

CCDC	1876722	
Crystal data		
Chemical formula	C_18_H_18_O_2_	
*M*_r_	266.32	
Crystal system, space group	Monoclinic, *P*2_1_/*c*	
Temperature (K)	110	
*a*, *b*, *c* (Å)	10.8006 (5), 8.7496 (3), 15.9339 (8)	
β (°)	107.431 (5)	
*V* (Å^3^)	1,436.62 (12)	
*Z*	4	
Radiation type	Mo *K*α	
μ (mm^−1^)	0.08	
Crystal size (mm)	0.42 × 0.30 × 0.22	
Data collection		
Diffractometer	SuperNova, Dual, Cu at zero, Atlas	
Absorption correction	Gaussian *CrysAlis* *PRO*, Agilent Technologies, Version 1.171.36.32 (release 02‐08‐2013 CrysAlis171.NET) (compiled Aug 2 2013,16:46:58) Numerical absorption correction based on Gaussian integration over a multifaceted crystal model	
*T*_min_, *T*_max_	0.343, 1.000	
No. of measured, independent and observed [*I* > 2σ(*I*)] reflections	11,350, 3,293, 2,708	
*R*_int_	0.026	
(sin θ/λ)_max_ (Å^−1^)	0.650	
Refinement		
*R*[*F* ^2 ^> 2σ(*F* ^2^)], *wR*(*F* ^2^), *S*	0.045, 0.111, 1.04	
No. of reflections	3,293	
No. of parameters	184	
H‐atom treatment	H‐atom parameters constrained	
Δρ_max_, Δρ_min_ (e Å^−3^)	0.35, −0.23	

### 3.2 Characterization of compound (**II**)

3′,5‐diallyl‐[1,1′‐biphenyl]‐2,4′‐diol (**II**): Mp 87–88°C; IR (neat, ν in cm^‐1^): 3,293, 3,080, 1,494, 1,429, 1,326, 1,241, 1,185, 1,050, 917, 822, 774; ^1^H‐NMR (600 MHz, CDCl_3_) δ 7.22 (m, 2H), 7.04 (dd, *J = *8.28, 1.86 Hz, 1H), 7.01 (m, 1H), 6.88 (dd, *J = *8.16, 2.04 Hz, 2H), 5.99 (m, 2H), 5.15 (m, 2H), 5.05 (m, 2H), 3.44 (d, *J = *6.36 Hz, 2H), 3.34 (d, *J = *6.66 Hz, 2H); ^3^C‐NMR (150 MHz, CDCl_3_) δ 153.94, 150.77, 137.81, 136.01, 132.28, 131.17, 130.25, 129.65, 128.85, 128.58, 127.75, 126.40, 116.96, 116.60, 115.63, 115.59, 39.43, 35.17.

**Figure 3:**
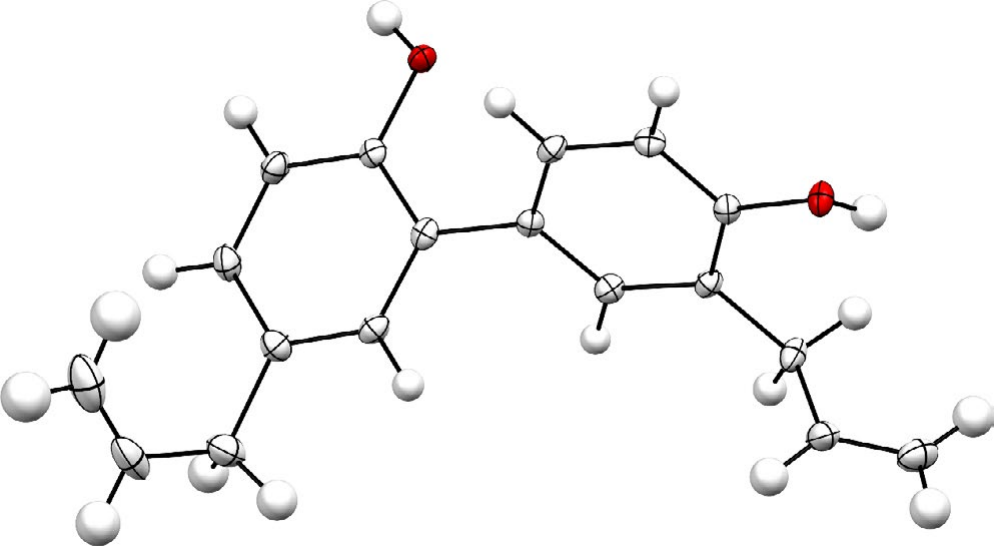
X‐ray crystallographic analysis of compound (**II**)

In FT‐IR spectrum, the broad peak at 3,293 cm^‐1^ identified the phenolic O–H stretching, whereas the presence of terminal double bonds was determined by 1D‐NMR experiments. APT and DEPT experiments revealed the presence of three aromatic methine carbons, one aliphatic methine carbon, two methylene carbons, and three quaternary carbons in the molecule. The structure of the compound (**II**) was further confirmed by X‐ray crystallographic analysis. The ORTEP projection of the compound (**II**) is presented in Figure 3.

The structure (**II**) was solved following the method stated earlier, and the details are shown in Table 2. The structure is ordered.

**Table 2:** X‐ray crystallographic details of the compound (**II**)

CCDC	1876723
Crystal data	
Chemical formula	C_18_H_18_O_2_
*M*_r_	266.32
Crystal system, space group	Monoclinic, *P*2_1_/*c*
Temperature (K)	110
*a*, *b*, *c* (Å)	12.5019 (4), 14.3062 (4), 7.8803 (3)
β (°)	93.885 (3)
*V* (Å^3^)	1,406.19 (8)
*Z*	4
Radiation type	Mo *K*α
μ (mm^−1^)	0.08
Crystal size (mm)	0.53 × 0.24 × 0.19
Data collection	
Diffractometer	SuperNova, Dual, Cu at zero, Atlas
Absorption correction	Gaussian *CrysAlis* *PRO*, Agilent Technologies, Version 1.171.36.32 (release 02‐08‐2013 CrysAlis171.NET) (compiled Aug 2 2013,16:46:58) Numerical absorption correction based on gaussian integration over a multifaceted crystal model
*T*_min_, *T*_max_	0.600, 1.000
No. of measured, independent and observed [*I* > 2σ(*I*)] reflections	10,879, 3,229, 2,762
*R*_int_	0.025
(sin θ/λ)_max_ (Å^−1^)	0.649
Refinement	
*R*[*F* ^2^ > 2σ(*F* ^2^)], *wR*(*F* ^2^), *S*	0.043, 0.103, 1.05
No. of reflections	3,229
No. of parameters	183
H‐atom treatment	H‐atom parameters constrained
Δρ_max_, Δρ_min_ (e Å^−3^)	0.29, −0.19

### 3.3 Characterization of compound (**III**)

(3*S*,3*aS*,8*S*,9*aS*,10*aR*,10*bS*,*E*)‐8‐hydroxy‐3,6,9*a*‐trimethyl‐3*a*,4,5,8,9,9*a*,10*a*,10*b*‐octahydrooxireno[2′,3′:9,10]cyclodeca[1,2‐*b*]furan‐2(3*H*)‐one or 2α‐hydroxydihydroparthenolide (**III**): Mp 220–221°C; IR (neat, ν in cm^–1^): 3,492, 1,760, 1,456, 1,229, 1,183, 1,028, 1,012, 806; ^1^H‐NMR (600 MHz, CDCl_3_) δ 5.20 (d, *J = *10.26 Hz, 1H), 4.81 (d, *J = *4.08 Hz, 1H), 4.44 (m, 1H), 3.96 (t, *J = *9.12 Hz, 1H), 3.33 (s, 1H), 2.82 (d, *J = *9.12 Hz, 1H), 2.37 (m, 2H), 2.31 (dd, *J = *11.82, 5.76 Hz, 1H), 2.17 (dd, *J = *12.72, 6.48 Hz, 1H), 1.98 (m, 2H), 1.80 (m, 1H), 1.63 (s, CH_3_), 1.19 (s, CH_3_), 1.13 (d, *J = *7.02 Hz, CH_3_); ^3^C‐NMR (150 MHz, CDCl_3_) δ 178.08, 134.16, 130.59, 81.81, 65.98, 65.23, 60.92, 50.91, 46.20, 41.98, 40.98, 28.86, 18.44, 17.60, 13.28.

**Figure 4:**
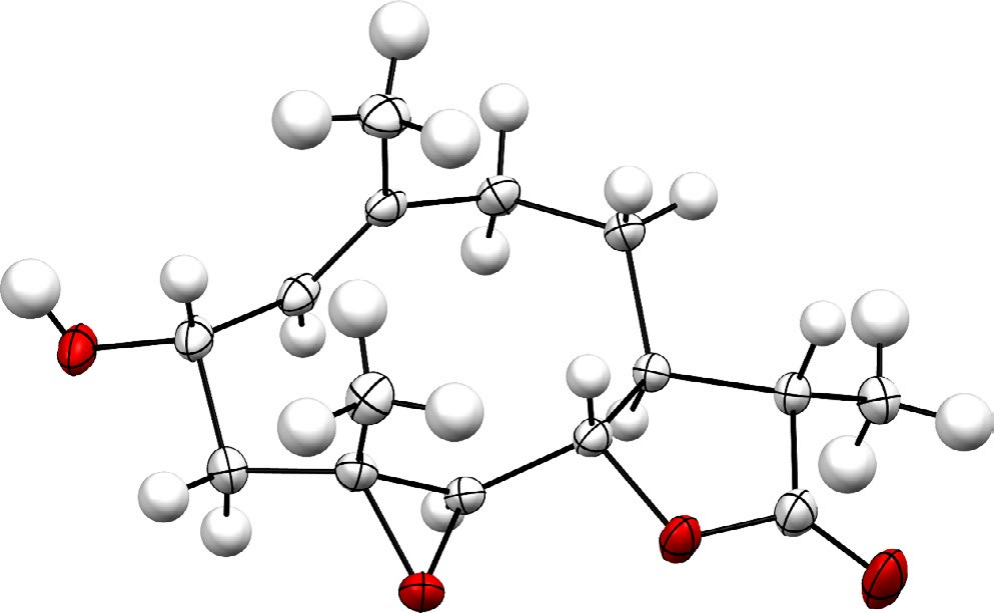
X‐ray crystallographic analysis of compound (**III**)

In FT‐IR spectroscopy, the alcoholic –OH hydrogen appeared at 3,492 cm^‐1^ and the lactone carbonyl appeared at 1,760 cm^‐1^. The presence of three methyl groups was confirmed by APT and DEPT experiments. The three methylene carbons appeared at δ 46.20, 40.98, and 28.86. The structure and stereochemistry of the compound (**III**) were determined by 2D‐NMR spectroscopy and further confirmed by X‐ray crystallographic analysis. The ORTEP projection of the compound (**III**) is presented in Figure 4.

The structure of (**III**) was solved following the method stated earlier, and the details are shown in Table 3. The structure is ordered and the absolute configuration was established by anomalous‐dispersion effects in diffraction measurements on the crystal, and the Flack and Hooft parameters refine to −0.08(6) and −0.08(5), respectively. The structure has chirality *S*, *S*, *S*, *S*, *R*, *R* at C1, C6, C7, C9, C10, and C11, respectively.

**Table 3:** X‐ray crystallographic details of the compound (**III**)

CCDC	1876724
Crystal data	
Chemical formula	C_15_H_22_O_4_
*M*_r_	266.32
Crystal system, space group	Orthorhombic, *P*2_1_2_1_2_1_
Temperature (K)	110
*a*, *b*, *c* (Å)	7.68084 (12), 11.44478 (17), 15.6640 (2)
*V* (Å^3^)	1,376.95 (3)
*Z*	4
Radiation type	Cu *K*α
μ (mm^−1^)	0.75
Crystal size (mm)	0.53 × 0.24 × 0.16
Data collection	
Diffractometer	SuperNova, Dual, Cu at zero, Atlas
Absorption correction	Analytical *CrysAlis* *PRO* 1.171.39.29c (Rigaku Oxford Diffraction, 2017) Analytical numeric absorption correction using a multifaceted crystal model based on expressions derived by Clark and Reid (1995)
*T*_min_, *T*_max_	0.782, 0.917
No. of measured, independent and observed [*I* > 2σ(*I*)] reflections	8,913, 2,704, 2,642
*R*_int_	0.018
(sin θ/λ)_max_ (Å^−1^)	0.616
Refinement	
*R*[*F* ^2^> 2σ(*F* ^2^)], *wR*(*F* ^2^), *S*	0.027, 0.073, 1.06
No. of reflections	2,704
No. of parameters	177
H‐atom treatment	H‐atom parameters constrained
Δρ_max_, Δρ_min_ (e Å^−3^)	0.20, −0.14
Absolute structure	Flack x determined using 1,100 quotients [(I+)‐(I‐)]/[(I+)+(I−)] (Parsons, Flack, & Wagner, 2013).
Absolute structure parameter	−0.08 (6)

### 3.4 GC‐MS studies of the oil

**Figure 5:**
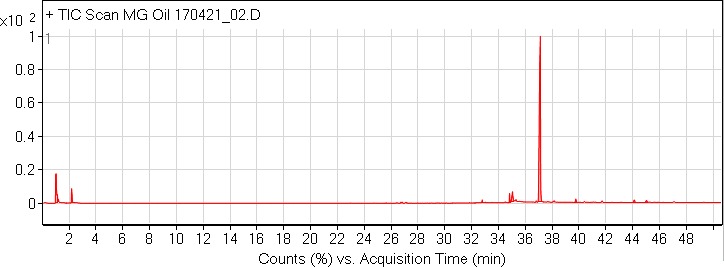
GC‐MS chromatogram of the oil isolated from *M. grandiflora* green seed cones

A dark red oil was isolated by eluting hexanes during column chromatography of the diethyl ether extract. GC‐MS analysis indicated the presence of eight compounds in the oil among which 5,5′‐diallyl‐2′‐methoxy‐[1,1′‐biphenyl]‐2‐ol (Compound **IV**, Figure 1) was found to be the major constituent (75.37%; RT = 37.112). The –OCH_3_ carbon appeared at δ55.53, and the presence of the two medicinally privileged phenylpropanoid skeletons was confirmed by FT‐IR and 1D‐NMR spectra. The compounds were identified based on retention time (RT), area under the curve (AUC), and mass spectral (m/z) analysis (Figure 5).

**Figure 6:**
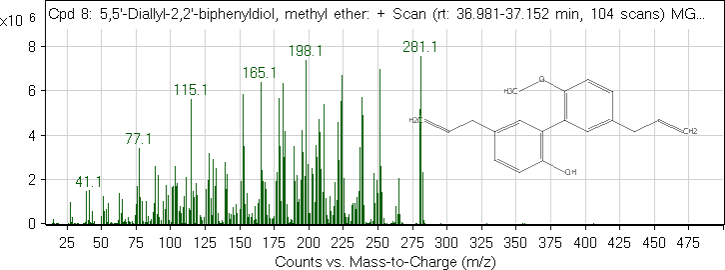
GC‐MS spectrum of the compound (**IV**)

**Figure 7:**
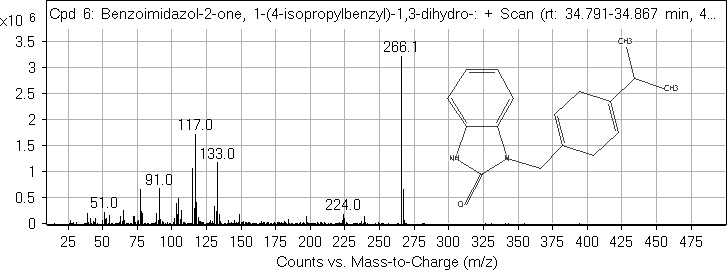
GC‐MS spectrum of the compound (**V**)

Aside compound (**IV**), other identified compounds are as follows: *Cis*‐vaccenic acid (4.14%; RT = 35.044), 1‐(*4*‐isopropylbenzyl)‐1,3‐dihydro‐2*H*‐benzo[*d*]imidazol‐2‐one (1.72%; RT = 34.822; compound **V**), heptacosane (0.98%; RT = 44.127), heneicosane (0.94%; RT = 39.759), 13‐methylheptacosane (0.86%; RT = 45.031), 1,1‐difluoroethane (0.83%; RT = 1.113), and *n*‐hexadecanoic acid (0.5%; RT = 32.786). The mass spectra of compound (**IV**) and (**V**) are shown in Figures 6 and 7, respectively.

In the next step, the druggability of the compounds (**I**–**V**, Figure 1) was validated following Lipinski's rule‐of‐five (RO5) (Lipinski, 2004; Lipinski, Lombardo, Dominy, & Feeney, 1997). Compounds **I**, **III**, and **V** showed no violation, whereas other two compounds (**II** and **IV**) showed only one violation each. Accordingly, all the five compounds can be considered as “drug‐like” molecules and semi‐synthetic products derived from these molecules (with appropriate chemical modification) have high promise to become effective drug(s). The druggability (druglikeness) of the compounds (**I**–**V**, Figure 1) was calculated by Molinspiration software is shown in Table 4.

**Table 4:** Druggability validation^a^ of the compounds **(I‐V)**

Compound	miLogP^b^	HBA^c^	HBD^d^	TPSA^e^	RB^f^	MW^g^	Violation (RO5)
**I**	4.80	2	2	40.46	5	266.34	0
**II**	5.00	2	2	40.46	5	266.34	1
**III**	1.10	4	1	59.06	0	266.34	0
**IV**	5.07	2	1	29.46	6	280.37	1
**V**	4.25	3	1	37.80	3	266.34	0

a Molinspiration property engine v2016.10.

b miLogP: Moriguchi octanol–water partition coefficient is based on quantitative structure‐LogP relationships, by using topological indexes.

c Hydrogen bond acceptor.

d Hydrogen bond donor.

e Total polar surface area.

f Number of rotatable bonds.

Molecular weight in Dalton.

## 4 CONCLUSION

In conclusion, five bio‐privileged molecules have been identified as the major components of the diethyl ether and ethanol fractions of *Magnolia grandiflora* green seed cones, and out of these, X‐ray crystallographic analyses of three compounds (**I**, **II**, and **III**) were successfully carried out. All the five compounds (**I**–**V**) discussed herein showed good to excellent in silico druggability. Some of these molecules may find application to open new frontiers in natural drug discovery research *via* appropriate chemical modification.

## CONFLICTS OF INTEREST

The authors do not have any conflicting interests.

## ETHICAL APPROVAL

This study does not involve any human or animal testing.

## INFORMED CONSENT

None.
